# Ancient DNA re-opens the question of the phylogenetic position of the Sardinian pika *Prolagus sardus* (Wagner, 1829), an extinct lagomorph

**DOI:** 10.1038/s41598-023-40746-w

**Published:** 2023-08-21

**Authors:** Valerio Joe Utzeri, Elisabetta Cilli, Francesco Fontani, Daniel Zoboli, Massimiliano Orsini, Anisa Ribani, Adriana Latorre, Andrey A. Lissovsky, Gian Luigi Pillola, Samuele Bovo, Giorgio Gruppioni, Donata Luiselli, Luca Fontanesi

**Affiliations:** 1 Department of Agricultural and Food Sciences, Division of Animal Sciences, University of Bologna, Viale Giuseppe Fanin 46, 40127 Bologna, Italy; 2 Department of Cultural Heritage, University of Bologna, Via degli Ariani 1, 48121 Ravenna, Italy; 3 Department of Chemical and Geological Sciences, University of Cagliari, Cittadella Universitaria SS 554, 09042 Monserrato, Italy; 4 Laboratory of Microbial Ecology, Istituto Zooprofilattico Sperimentale delle Venezie, Viale dell’università 10, 35120 Legnaro, Italy; 5 A.N. Severtsov Institute of Ecology and Evolution of the Russian Academy of Sciences, Moscow, Russia

**Keywords:** Evolution, Palaeontology, Phylogenetics

## Abstract

Palaeogenomics is contributing to refine our understanding of many major evolutionary events at an unprecedented resolution, with relevant impacts in several fields, including phylogenetics of extinct species. Few extant and extinct animal species from Mediterranean regions have been characterised at the DNA level thus far. The Sardinian pika, *Prolagus sardus* (Wagner, 1829), was an iconic lagomorph species that populated Sardinia and Corsica and became extinct during the Holocene. There is a certain scientific debate on the phylogenetic assignment of the extinct genus *Prolagus* to the family Ochotonidae (one of the only two extant families of the order Lagomorpha) or to a separated family Prolagidae, or to the subfamily Prolaginae within the family Ochotonidae. In this study, we successfully reconstructed a portion of the mitogenome of a Sardinian pika dated to the Neolithic period and recovered from the Cabaddaris cave, an archaeological site in Sardinia. Our calibrated phylogeny may support the hypothesis that the genus *Prolagus* is an independent sister group to the family Ochotonidae that diverged from the *Ochotona* genus lineage about 30 million years ago. These results may contribute to refine the phylogenetic interpretation of the morphological peculiarities of the *Prolagus* genus already described by palaeontological studies.

## Introduction

Studies on ancient DNA (aDNA) of extinct animal species have made possible to dig into the past and complement classical palaeontological approaches to reconstruct evolutionary trajectories at an unprecedent level^1–6^. In particular, mitochondrial DNA (mtDNA) represents a useful tool to understand phylogeny and population dynamics due to its abundance in ancient remains with respect to the nuclear genome, high mutation rate and nearly neutral mode of evolution^7–12^.

A few extinct animal species from Mediterranean regions have been characterised at the DNA level thus far, providing information on the unique evolutionary history of isolated and endemic species^3,4,7,13–16^. An interesting case study is based on one Sardinian extinct species. Palaeogenomic data related to the enigmatic extinct Sardinian dhole, *Cynotherium sardous* Studiati, 1857, demonstrated that this unique canid species, that populated the island till the end of the Late Pleistocene, represents a separate taxon from all other living canids and became genetically isolated from other mainland canid lineages approximately 500–300 kya (Middle Pleistocene)^4^.

Another enigmatic and iconic extinct species that populated Sardinia and Corsica is the Sardinian pika *Prolagus sardus* (Wagner, 1829)^17–19^. The genus *Prolagus* Pomel, 1853, which belongs to the order Lagomorpha, is documented by several species which lived during the Neogene and the Quaternary^19–21^. Fossils pertaining to this genus have been identified in many sites of Europe, North Africa, and Anatolia. The evolutionary history and the anatomy of *Prolagus* have been discussed by several authors who traditionally considered this genus as a peculiar member of the family Ochotonidae Thomas, 1897^20–29^, that currently includes only the living pikas, all classified within the genus *Ochotona* Link, 1795. A few other authors, however, have proposed that all extinct *Prolagus* species belong to the separate family Prolagidae Gureev, 1964, or alternatively to a subfamily of Ochotonidae, Prolaginae^23–28^. The lack of the third lower molar (M_3_) is one of the main anatomic elements that differentiates *Prolagus* species from the genus *Ochotona*^20,30^. *Prolagus* is thought to have been anagenetically derived from the monospecific genus *Piezodus* Viret, 1929, a taxon from the Oligocene-Lower Miocene of Europe, which differs from *Prolagus* for the presence of rooted cheek teeth and metaflexids, and the lack of protoconulid in the third lower premolar^20,21,31,32^. *Prolagus* species are known to be present in Mediterranean insular environments as documented by the taxa retrieved in the Gargano palaeo-archipelago (Late Miocene?—earliest Pliocene) and in the Sardinia-Corsica Block (Late Pliocene—Holocene). In Corsica and Sardinia, the genus *Prolagus* is represented by an anagenetic lineage that includes three endemic taxa: *Prolagus* aff. *figaro* (early Late Pliocene of Capo Mannu D1), *Prolagus figaro* López-Martínez, 1975 (latest Pliocene?/earliest Pleistocene—late Early Pleistocene) and *Prolagus sardus* (Wagner, 1829) (Middle Pleistocene—Holocene)^17,20,32–36^.

Among the fossil remains of Quaternary vertebrates of Sardinia, those of *Prolagus sardus* are certainly the most abundant^37–44^. It is likely that *P. sardus* was a prey of several carnivores (canids and mustelids) and prey birds of the Quaternary insular palaeo-ecosystem of Corsica and Sardinia. In particular, it seems possible that the endemic canid *Cynotherium sardous* was specialised to hunt *P. sardus*^45,46^. Burnt remains collected in several archaeological sites documented that *P. sardus* was regularly hunted and eaten by the first communities of modern humans who colonised the Sardinia-Corsica Block^39^. This lagomorph was certainly present in Sardinia until the Iron Age (^14^C age 2760 cal BP according to the site of Su Guanu cave) and in Corsica possibly until the Roman era (between 2343 BP and the sixth century CE according to the site of Castellu)^37–39,47,48^. The extinction of *P. sardus* is however probably linked to the introduction of new predators and ecological competitors in the insular ecosystem. The transmission of pathogens by introduced species such as rats and hares cannot be excluded as potential alternative events or co-events that led to the extinction of this species^37–39^.

In this study, we report the retrieval of sequences from the mitochondrial genome of *P. sardus* derived from a radiocarbon-dated bone specimen collected in a Sardinian archaeological site. Phylogenetic reconstructions based on these DNA sequences may suggest that the extinct *Prolagus* genus could be better placed in an independent sister group to the family Ochotonidae.

## Results

### Characterisation of the *Prolagus sardus* bone

**Figure 1:**
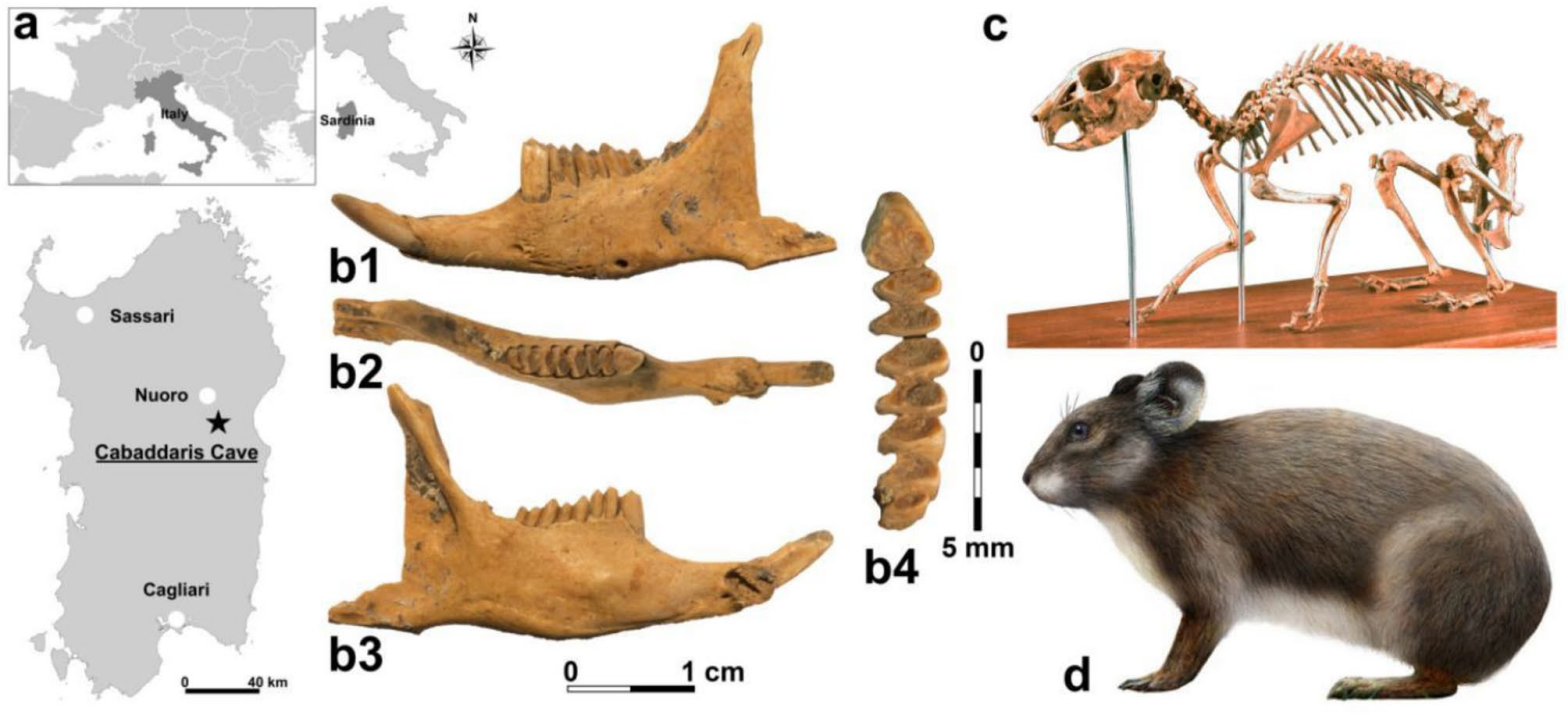
(**a**) Geographic location of the Cabaddaris Cave (Sardinia, Italy); (**b**) left hemimandible of *Prolagus sardus* (PR6 bone) used for DNA extraction and radiocarbon dating, from different perspectives (**b**_**1**_: buccal, **b**_**2**_: occlusal, **b**_**3**_: lingual and **b**_**4**_: occlusal view of the cheek teeth); (**c**) composite skeleton of *P. sardus* (Museo Sardo di Geologia e Paleontologia “D. Lovisato”, University of Cagliari); (**d**) palaeo-artistic reconstruction of *P. sardus* (drawing by D. Zoboli).

The analysed bone (specimen n. PR6) was an incomplete left hemimandible belonging to a mature Sardinian pika collected at Cabaddaris cave (Sardinia, Italy; Fig. 1). The hemimandible dimensions and the dentognatic features, such as the general features of P_3_, the absence of M_3_, and the presence of three lobes in M_2_, clearly allowed to assign the bone to *P. sardus* and to exclude all extinct and living Sardinian lagomorphs.

Radiocarbon dating conducted with the Accelerator Mass Spectrometry (AMS) method indicated a calibrated range from 7575 to 7431 cal BP with a probability of 95.4% (Supplementary material Fig. S1). This result attributed the analysed bone to the Neolithic (from 6000 to 4000 BCE).

### Ancient DNA from *Prolagus sardus*

The most sensitive and cutting-edge protocols for aDNA analysis were applied and the efficiency of the analysis was evaluated in each step. More than 32 million paired-end reads were generated from specimen PR6, but only an extremely low fraction of all obtained sequences (0.008%) mapped against the *Ochotona curzoniae* reference mitochondrial genome (accession number: NC_011029.1), indicating poor conservation of aDNA of this specimen.

Sequences that mapped to the *O. curzoniae* mitochondrial reference genomes reported typical patterns of degraded DNA, such as short length of the fragments (40 bp on average) and increasing frequency of C > T transitions at the most extreme positions of every read (Supplementary material Fig. S2). Considering the results of the alignment of these reads, the highest fraction of reads was mapped on the *O. curzoniae* (NC_011029.1), *O. princeps* (NC_005358.1) and *Oryctolagus cuniculus* (NC_001913.1) mitochondrial genomes, with 2721, 2608 and 2535 unique mapped reads (after filtering for duplicates), respectively.

A total of 3393 bp was obtained with clear ancient DNA damage patterns, corresponding to a 19.8% of the complete mitochondrial genome of *P. sardus*, if referred to the modern mitochondrial reference sequence of the ochotonid *O. curzoniae* (17,131 bp). The distribution of the obtained sequences along this reference mitochondrial genome is shown in Supplementary material Fig. S3.

**Figure 2:**
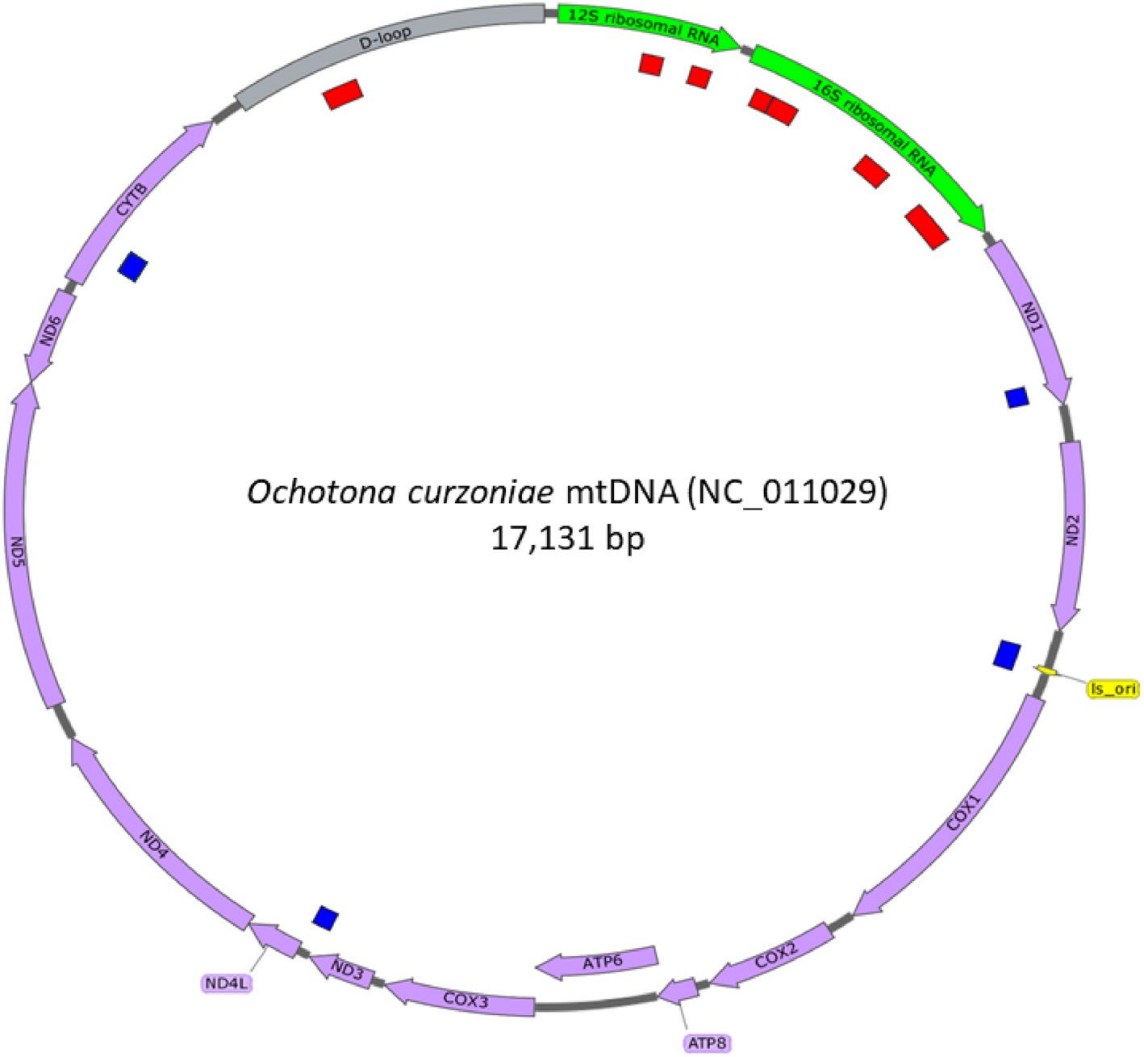
*Prolagus sardus* mtDNA consensus regions (inner circle) aligned against the *Ochotona curzoniae* complete mtDNA sequence. Only regions longer than 100 were reported here. Regions in red correspond to *P. sardus* mtDNA sequences used for phylogenetic analyses, instead blue regions were not considered. To simplify the graphical representation, tRNA features were not included in the reference sequence.

The alignment of reads to the *O. curzoniae* reference mtDNA sequence produced a total of 51 consensus regions, of which 12 were longer than 100 bp (Supplementary material Table S1). In particular, combining sequence information, for five mitochondrial genes and for the D-loop, sequence data was longer than 100 bp (Supplementary material Table S2). Figure 2 shows the reconstruction of *P. sardus* mtDNA contigs that matched the corresponding regions of the *O. curzoniae* mtDNA sequence.

### Phylogenetic relationships of *Prolagus sardus* and divergence time

**Figure 3:**
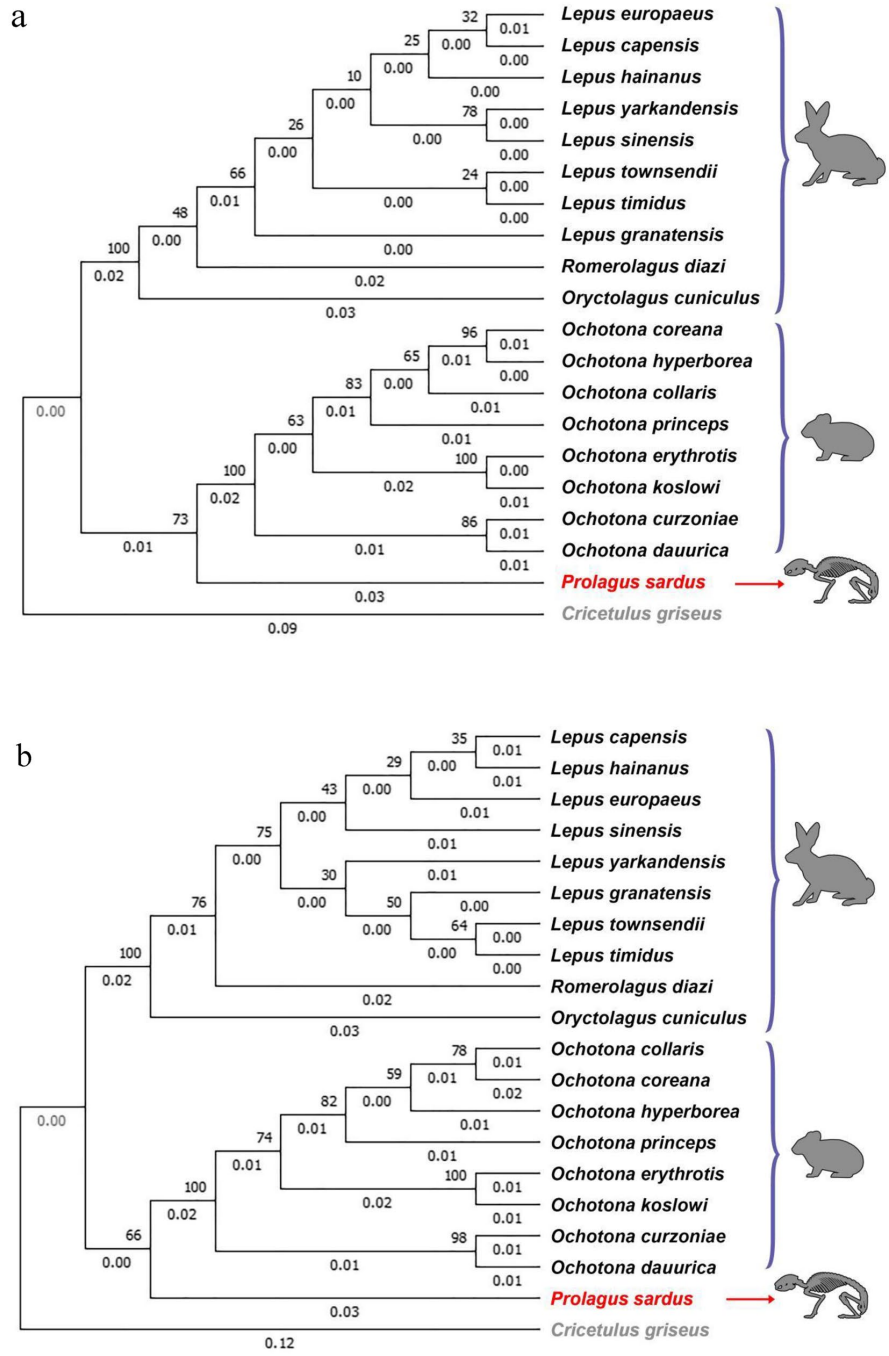
Maximum-likelihood trees obtained with: (**a**) ribosomal mtDNA contig sequences (16S + 12S); (**b**) D-loop and ribosomal contig sequences (D-loop + 16S + 12S). Node numbers represent confidence values. Branch lengths are proportional to the number of substitutions per site.

Phylogenetic trees were constructed using the obtained sequence information from *P. sardus* and from 18 mitochondrial genomes of extant lagomorph species (eight from the Ochotonidae and ten from Leporidae). The mitochondrial genome of the rodent species *Cricetulus griseus* was used as outgroup (Supplementary material Table S3). To avoid biases derived from too short, fragmented and non-informative mtDNA regions, phylogenetic analyses were carried out with alignments including the informative regions of the 12S, 16S and D-loop sequences or from a combined sequence including only the 12S and 16S regions. All considered coding sequences did not have any frameshift mutations (insertions/deletions) or nonsense mutations (stop codons). Including other sequences, results did not change (data not shown). In all the reconstructed phylogenetic trees, *P. sardus* was an outgroup taxon to all the Ochotonidae, indicating that it may represent a sister taxon of the ochotonid group (Fig. 3). Maximum Likelihood inference trees of partial mitochondrial genome of the *P. sardus* bone-derived sequences indeed established unequivocally that this extinct species is more closely related to the Ochotonidae clade than to the Leporidae clade (Fig. 3). In the phylogenetic analysis, the *Prolagus-Ochotona* clade is recovered with 66% and 73% maximum likelihood bootstrap support in the alignment with or without the D-loop sequence, respectively (Fig. 3). Nodes relative to the basal clusters of the two extant families of lagomorphs (Ochotonidae and Leporidae) are strongly supported with 100% bootstrap values as expected, and the basal node for the *Lepus* genus was one of the highest values inside the group of Leporidae (66%), as well as the basal node of 63% clustered the root of the *Ochotona* group.

**Figure 4:**
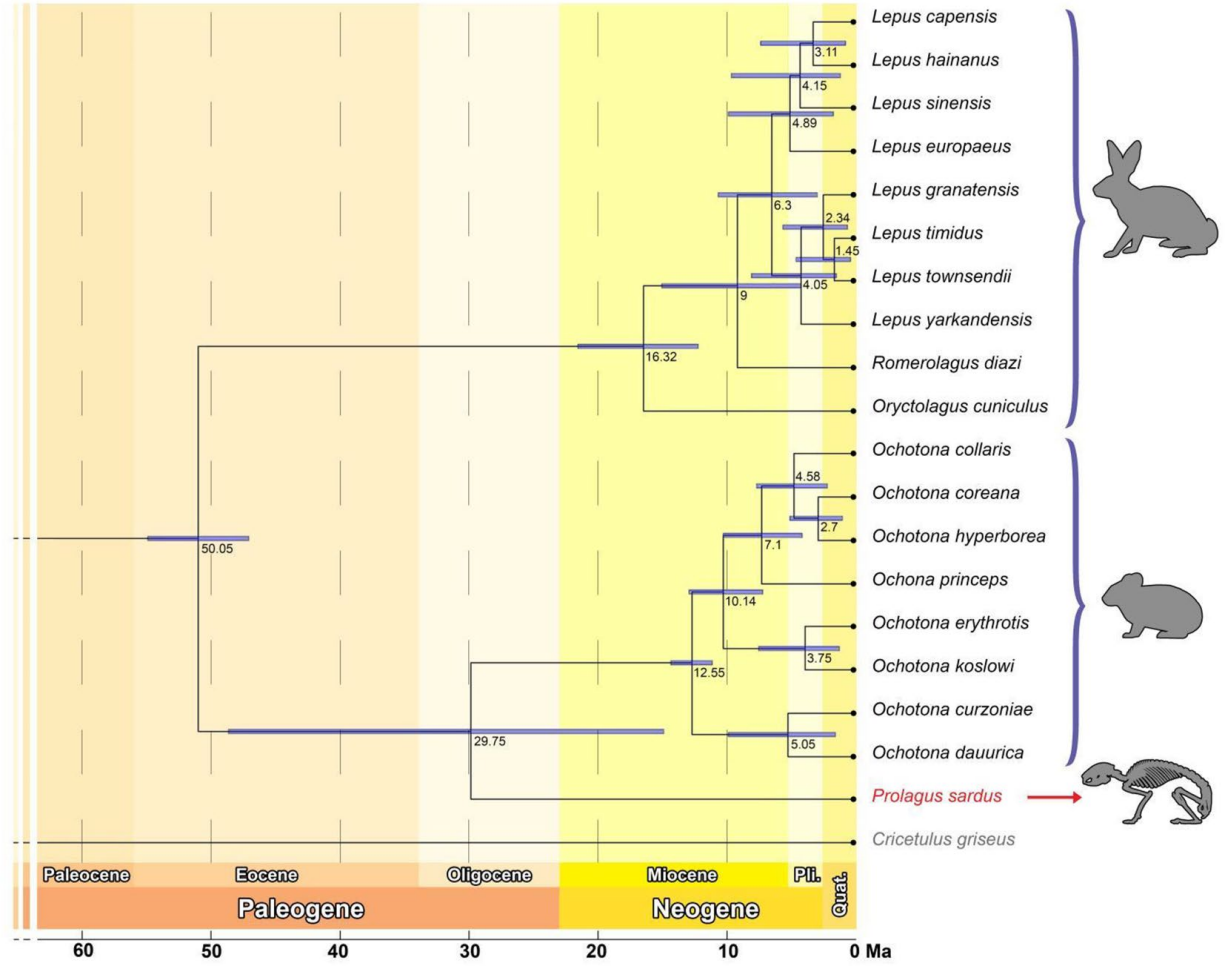
Divergence dates and 95% confidence intervals resulting from the analysis of the ~ 51 Ma rooted model (partial D-loop + partial 12S + partial 16S dataset) of the Bayesian relaxed molecular dating method implemented in BEAST2.

Molecular clock analyses of the mitogenomic data obtained from the *P. sardus* indicated that it diverged from the *Ochotona* lineage about 30 Ma (Fig. 4; Supplementary material Fig. S4). Our divergence estimations between the *Prolagus* and the *Ochotona* lineages match the estimates derived from previous palaeontological studies^49–51^.

## Discussion

Here we present the first evidence of DNA conservation from a bone of a Sardinian pika *Prolagus sardus*, dated to the Neolithic period, and recovered in the archaeological site of the Cabaddaris cave, in Sardinia. The radiocarbon dating of the analysed bone was in line with the expected raw estimation age of the remains in this cave based on previous archaeological investigations of the site.

The authenticity of the sequence data was evidenced by clean negative controls and DNA-damage patterns, which cast no doubts about the ancient origin of the retrieved sequence information. The low fraction of aDNA assigned to *P. sardus* that was possible to recover from this specimen could, however, confirm the general difficulties in retrieving well preserved DNA from ancient remains of the hot Mediterranean basin, if compared to other colder environments, such as permafrost^12,52,53^. In these conditions, therefore, many more copies of the mitochondrial genome than those of the nuclear genome might be usually retrieved from ancient bones. Considering that all *P. sardus* sequences matching mtDNA coding regions used in the phylogenetic analyses did not include any frameshift mutations or stop codons that would have disrupted the open reading frames, we can reasonably exclude that they could belong to mitochondrial DNA sequences present in the nuclear genome (NUMTs)^12,54,55^ and that, therefore, what we retrieved could be considered as derived from the true ancient mtDNA of *P. sardus*.

In general, by mapping reads against several lagomorph species of which *Ochotona curzoniae*, *O. princeps* and *Oryctolagus cuniculus* represent those with the highest fraction of hits, we recovered ~ 20% of the mitochondrial genome of *P. sardus.* Although the retrieved sequence was just about one fifth of the total expected mitochondrial genome, the ancient sequence data enabled the first molecular phylogenetic placement of this extinct species, as also obtained for other species in previous studies that used partial mtDNA sequence information^9,12^.

Only two extant families are recognised in the order Lagomorpha: Leporidae, which includes the rabbits, the hares, and the jackrabbits; Ochotonidae, which includes the pikas, all grouped in the monophyletic *Ochotona* genus^28,56–58^. These two families diverged about 51.4 Ma^59–61^. However, there is no commonly accepted consensus of the scientific community regarding the intraordinal distinction of the order Lagomorpha when the extinct groups are considered^20,62–66^. Conversely, several extinct families of Lagomorpha have been described, including †Paleolagidae Dice, 1929, †Mytonolagidae Burke, 1941, †Desmatolagidae Burke, 1941, †Prolagidae Gureev, 1962, †Mimolagidae Erbajeva, 1986, and †Strenulagidae Averianov and Lopatin, 2005^62^. In addition, various intraordinal classifications of the order Lagomorpha were proposed by other authors^23,67^. The family Prolagidae groups pika-like lagomorphs reported from the Oligocene-lowermost Miocene to the Holocene fossil records of the peri-Mediterranean area.

Phylogenetic analyses that we carried out with the obtained *P. sardus* sequences were performed with a dataset of corresponding 12S, 16S and D-loop sequences of mtDNA from modern species of Lagomorpha from different continents, using the mitochondrial genome of a rodent species as outgroup. As expected, the phylogenetic analysis supported the nodes of the two extant families with strong values for both Leporidae and Ochotonidae. In addition, within the Ochotonidae family, fine phylogenetic relationships were in line with previous studies of this group of mammals based on cytochrome b and ND4 mtDNA genes^70^, suggesting that partial mtDNA sequence used in this study did not miss phylogenetic microstructures and relationships within the order.

The phylogenetic trees obtained with and without the D-loop region were consistent in supporting the position of *P. sardus* in a sister clade to the Ochotonidae family. The *Prolagus-Ochotona* clade was recovered with good statistical support in both analyses (66% and 73% of bootstrap in the two phylogenetic reconstructions, i.e. with or without the D-loop sequence, respectively). In general, the node values increased with the addition of the D-loop sequence in the alignment, for all divergence nodes while this value decreased for the node of the *P. sardus* lineage. This might be mainly attributable to the different mutation ratios present in this hypervariable region^71,72^.

Bayesian molecular dating implemented in BEAST highlighted that the *P. sardus* diverged from *Ochotona* spp. about 30 Ma (CI: 15–59 Ma). Therefore, our molecular phylogenetic analyses support an independent evolutionary scenario for the Oligocene-Holocene *Piezodus-Prolagus* lineage as previously suggested by palaeontological data. Furthermore, the available data support the hypothesis to include the genus *Prolagus* in the independent family Prolagidae. Our results can therefore suggest that a new taxon including *Prolagus* may better explain the molecular diversity found and, in turn, the morphological diversity described by palaeontological studies, in line to what was suggested by some authors using only palaeontological information^23–28^.

This study provides the first molecular systematic characterisation of *P. sardus*, paving the way for its correct phylogenetic assignment. Further studies are needed to obtain a complete mtDNA sequence of this extinct species able to confirm the inferred molecular dating. More *P. sardus* specimens should be also investigated to retrieve aDNA from more individuals and understand the intra-specific diversity of this species. It could be also interesting to evaluate the evolutionary trajectory of this species over time by analysing aDNA of other specimens dated over a wide time window and retrieved both in Sardinia and Corsica.

## Methods

### *Prolagus sardus* bone sample

One mandible of *Prolagus sardus* (PR6) excavated from the archaeological site of the Cabaddaris cave (Lat. 40°08′46′′, Long. 3°00′45′′; Supramonte di Orgosolo, Nuoro) in Sardinia (Italy) was used in this study (Fig. 1). The sample was retrieved in an archaeological site of the Pre-Nuragic period of Sardinia. However, since no clear stratigraphic data were available, the sample was sent for radiocarbon dating. The bone included the complete *corpus mandibulae* and part of the *ramus mandibulae*. The *processus condylaris* and the proximal part of the *processus coronoideus* were not preserved. The dentition was complete, and the lower incisor and the cheek teeth (P_3_-M_2_) were well preserved. No remains of other lagomorphs were found in the Cabaddaris cave in association with this mandible and the human artifacts. The sample was provided by the Soprintendenza Archeologia, Belle Arti e Paesaggio per le Province di Sassari e Nuoro. The Soprintendenza represents the territorial branches of the Government, responsible for the management and entrustment of cultural heritage in Italy.

Biomolecular (e.g. aDNA, paloeoproteomics) and physical-chemical (e.g. isotopes, radiocarbon dating) analyses, provide fundamental information and data, however, they often implicate destructive or micro-destructive approaches. To preserve precious ancient remains, it is important to apply, when possible, appropriate methods of digital acquisition, with the aim to allow future analyses or to leave the possibility of having access to a reproduction of the remain for museum purposes. Thus, microphotogrammetry was used to obtain a 3D graphical model of the mandible before DNA extraction and radiocarbon dating (Fig. 1). We performed a total of 85 high resolution photos using the camera Olympus OM-D E-M10 MarkII with Olympus M. Zuiko digital ED 12–50 mm lenses at 0.36x (Macro Mode). The images were subsequently acquired and processed using the software Agisoft Photoscan PRO (Agisoft, St. Petersburg, Russia) by merging dense clouds from five different chunks and by manually refining the model obtained to a total of 3000 points. The 3D model here obtained is available at the MorphoSource repository (https://www.morphosource.org/concern/media/000494910).

### Radiocarbon dating of the bone sample

To generate radiocarbon dating for the remain of *Prolagus sardus*, we weighed the jaw with a precision laboratory balance (0.9 g). The sample was sent to the Centre of Dating and Diagnostic, Department of Mathematics and Physics (CEDAD) laboratory, University of Salento (Italy) for radiocarbon dating, that was obtained with the CEDAD Accelerator Mass Spectrometry (AMS), following the methods previously described^73,74^. Reference samples of known oxalic acid concentration provided by the National Institute of Standard and Technology (NIST) were used as a quality control of the results. Radiocarbon dating was then calibrated in calendar age using OxCal Ver. 3.10 software based on INTCAL20 atmospheric data^75^.

### DNA extraction and sequencing

The PR6 sample, represented by an almost complete left jaw (Fig. 1), was processed in the Ancient DNA Laboratory of the University of Bologna (Department of Cultural Heritage, Ravenna, Italy). Strict criteria established for aDNA analyses were followed to ensure the authentication of the data and avoid contaminations^76–78^. In fact, disposable coverall suits, double pair of gloves, boots, face masks and plastic face shields were worn during the handling of samples. The worktop and instruments were regularly cleaned after each experiment with DNA Exitus Plus solution (AppliChem GmbH, Darmstadt, Germany) or ~ 5% commercial NaClO and exposed for 30 min to ultraviolet radiation at 254 nm. Moreover, DNA-free reagents were used, and negative controls were processed for each batch of samples. In addition, PCR reactions and post-PCR procedures were carried out in a physically isolated area.

The teeth, represented by one incisor and the cheek teeth, all still encased into the alveoli, were sampled and pooled together. Due to the small size of the teeth, it was not possible to sample only the cementum part. They were decontaminated by spraying 1.5–2.0% sodium hypochlorite on the surface of the teeth, and then a clean paper towel, rinsed with DNA-free water, was used to remove the bleach. They were subsequently air-dried for a minimum of 20 min under ultraviolet (UV) light (254 nm wavelength) at a distance of ~ 25 cm from the bulb. Then, they were crushed in a mortar and ~ 100 mg of powder was recovered. The DNA extraction was performed using a modified version of a silica-based column protocol, by adding a pre-digestion step^79–81^. One extraction negative control was included in the process. Briefly, the digestion buffer was composed by 0.45 M EDTA, 0.25 mg/mL proteinase K, 0.05% Tween, the binding buffer was the PB Buffer (Qiagen, Hilden, Germany) and silica columns were the ones from High Pure Viral Nucleic Acid Large Volume kit (Roche, Basel, Switzerland). Two washing steps with PE Buffer (Qiagen) were performed and finally the DNA was eluted in 50 µL of EB Buffer (Qiagen) and quantified with Qubit instrument (Thermo Fisher Scientific, Waltham, MA, USA) and High Sensitivity Assay dsDNA kit (Thermo Fisher Scientific).

We first attempted a multiplexed mtDNA sequence capture protocol^82^ with home-made baits obtained from long range PCR of *Ochotona princeps* DNA but the enrichment of ancient mtDNA was unsuccessful (data not shown). Then we proceeded with a shotgun approach. A total of 20 µL of the eluted material was converted in single strand libraries^83^ along with a library as negative control. The optimal number of PCR cycles for indexing was determined by Real-Time amplification on ABI 7500 PCR System (Thermo Fisher Scientific). Indexing amplification was performed by splitting 48 µL of each library in two 50 µL reactions for 18 cycles, using a dual indexing approach and AmpliTaq Gold 360 Master Mix (Thermo Fisher Scientific). The amplified libraries were purified using 0.9X AMPure SPRI-beads (Beckman Coulter Life Sciences, Brea, CA, USA) and quantified on the Bioanalyzer (Agilent Technologies, Santa Clara, CA, USA) using the DNA1000 kit (Agilent Technologies). The indexed libraries from these and other ancient samples were then pooled in equimolar amounts and sequenced on a lane of an Illumina HiSeq X platform run (Illumina, San Diego, CA, USA) for 75 × 2 paired-end sequencing, generating more than 32 million paired-end reads.

### Bioinformatic analyses

Raw data were initially investigated with FastQC^84^ to evaluate the performance of the sequencing process. Sequences were processed with Paleomix^85^ by aligning paired-end reads to the complete versions of seven mitochondrial genomes of different species: *Sciurus vulgaris* (accession number: NC_002369.1), *Lepus capensis* (GU937113), *Mus musculus* (NC_005089.1), *Ochotona curzoniae* (NC_011029.1), *O. princeps* (NC_005358.1), *Oryctolagus cuniculus* (AJ001588.1) and *Homo sapiens* (NC_007092.1). Briefly, adapters were trimmed with lenient parameters using AdapterRemoval (maximum number of mismatches allowed when trimming barcodes ≤ 3, reads discarded if length < 20). To improve mappings of ancient reads at the ends of the circular reference genomes, we generated elongated versions (elongation value k = 500) of the mitogenomes using the CircularGenerator tool implemented in EAGER^86^. The collapsed reads were initially aligned to the elongated references using BWA backtrack algorithm, with minimum quality of reads to be aligned ≥ 20 and seed region use disabled^87^. Preliminary BAM files were then realigned to the standard version of the reference mitogenomes using the CircularMapper tool from EAGER. Duplicates were removed with PCRDuplicates on Paleomix^85^. The authenticity of data was finally estimated by computing frequencies of C > T along the 5’ and 3’ ends of the reads with mapDamage2^88^ and by observing the number of reads aligning to the human reference genome. Sequences with a minimum depth of 3X were subsequently manually inspected with Integrative Genomics Viewer (IGV)^89^ and used for further analyses. Reconstruction of the *P. sardus* mtDNA contigs was obtained with GenomeVX^90^ using *O. curzoniae* mtDNA reference sequence (accession number: NC_011029.1; 17,131 bp).

### Phylogenetic analyses

*Prolagus sardus* sequences obtained in this study were aligned with 18 previously published 12S, 16S and D-loop regions of mtDNA from modern Lagomorpha species of different continents and one Rodentia species (*Cricetulus griseus*) as outgroup. The list of species and the relative information are shown in Supplementary material Table S1. The alignment was obtained using MEGA X^91^, with the tool ClustalW. Phylogenetic analyses were carried out on phylogenetically informative regions of the mtDNA genome (12S, 16S and D-loop) that have been previously established for the two lagomorph families, Leporidae and Ochotonidae^60,71,92^. Two phylogenetic analyses were performed using the following sequence information: (i) a contig obtained joining three partial ribosomal sequences (two contigs of the 16S gene regions of 259 bp and 154 bp, respectively; and 1 contig of the 12S gene region of 118 bp) for a total of 531 bp of *P. sardus* mtDNA sequence: (ii) to this contig, a portion of the D-loop sequence of 200 bp was added to obtain another combined contig of 731 bp of the *P. sardus* mitochondrial genome sequence. Maximum likelihood trees were obtained using RAxML-NG^93^, providing, for each dataset, a starting Neighbour-joining (NJ) tree based on the Jukes and Cantor (JC) model substitution matrix^94^, with 1000 bootstraps, as suggested by the tool “Models”, implemented in MEGA X^91^. The most suitable model (JC) was chosen since it showed the best probability with and without outgroup (LnL -3021.9; LnL -20,647.8).

### Molecular dating

Bayesian estimation of the divergence time between *P. sardus* and other Lagomorpha taxa was obtained with BEAST 2.5^95^ using alignments of partial sequences of D-loop, 16S and 12S mtDNA regions already used for the phylogenetic analyses. Bayesian estimation analyses were performed including species of the two families of Lagomorpha order (Leporidae and Ochotonidae) and one outgroup of the order Rodentia (*Cricetulus griseus*, accession number NC_007936.1). BEAST 2.5 was run by assuming a combination of parameters including a JC69, Gamma4 substitution model, a relaxed uncorrelated lognormal molecular clock, and a Yule process of speciation^91,96^. The following three calibration points were defined considering a Log normal distribution: (i) the divergence (most recent common ancestor, MRCA) between Ochotonidae and Leporidae families (51.4 Ma, CI 49.6–53.1)^59–61^; (ii) the divergence node of *Ochotona* group (12.2 Ma, CI 10.9–19.4)^60,61^; (iii) the node between *Romerolagus diazi* and the *Lepus* genus (17.6 Ma, CI 13.4–23.7)^92,97^. Analyses were based on two independent Markov Chain Monte Carlo (MCMC) chains, each consisting of 10,000,000 chains. Data were collected every 1,000 points, and the burn-in was set to 10%. The chain convergence and effective sampling size (ESS) values higher than 200 were evaluated with Tracer v. 1.5 (Available: http://tree.bio.ed.ac.uk/software/tracer). TreeAnnotator v. 2.6.7 (Available: https://beast.community/treeannotator, Accessed 2022 Gen 1) and Figtree v. 1.4.4 (Available: http:// http://tree.bio.ed.ac.uk/software/figtree/, Accessed 2021 Dec 1) were used to annotate and illustrate the final trees.

An independent clock analysis was carried out by applying the RelTime method available in the software MEGA X^91,98,99^. Branch lengths were calculated using the maximum likelihood (ML) method and the Jukes-Cantor substitution model was applied^100^. A Log-normal distribution calibration type setting, with two calibration constraints (Leporidae-*Ochotona* divergence node and *Ochotona* genus basal node) was applied with the same timing used for BEAST 2.5 analyses. Tao et al.^99^ method was used to set minimum and maximum time boundaries on nodes for which calibration densities were provided and confidence intervals were computed with the same method^100^. Bars around each node represent 95% confidence intervals. A discrete Gamma distribution was used to model evolutionary rate differences among taxa. The geological time scale followed Gradstein et al.^101^

### Supplementary Information

Supplementary Information.

## Data Availability

The sequencing dataset generated and analysed during the current study is available in the EMBL-EBI European Nucleotide Archive (ENA) repository (http://www.ebi.ac.uk/ena) under the study accession PRJEB57248. The 3D model of the sample is available at the MorphoSource repository (https://www.morphosource.org/concern/media/000494910).
