# Supplementary material for: Ancient DNA re-opens the question of the phylogenetic position of the Sardinian pika Prolagus sardus (Wagner, 1829), an extinct lagomorph
Source: Sci Rep. 2023 Aug 21;13:13635. doi: 10.1038/s41598-023-40746-w (PMC10442435; doi:10.1038/s41598-023-40746-w)

Supplementary materials

Ancient DNA re-opens the question of the phylogenetic position of the Sardinian pika *Prolagus sardus* (Wagner, 1829), an ex-tinct lagomorph

Valerio Joe Utzeri, Elisabetta Cilli, Francesco Fontani, Daniel Zoboli, Massimiliano Orsini, Anisa Ribani, Adriana Latorre, Andrey A. Lissovsky, Gian Luigi Pillola, Samuele Bovo, Giorgio Gruppioni, Donata Luiselli, and Luca Fontanesi.

Table S1. Detailed information on the *Prolagus sardus* mtDNA sequences obtained in this work. A total of 51 contigs were obtained and 12 of these contigs were >100 bp.

**Region[1]**	**Range region on NC_011029.1**	**Length (bp)**
12S	71..1034	37
12S	71..1034	148
12S	71..1034	60
**12S**	**71..1034**	**118**
12S	71..1034	44
12S	71..1034	61
12S	71..1034	112
12S	71..1034	59
16S	1103..2669	104
**16S**	**1103..2669**	**154**
16S	1103..2669	61
16S	1103..2669	176
16S	1103..2669	42
16S	1103..2669	37
**16S**	**1103..2669**	**259**
16S-tRNA_Leu-ND1	1103..2669-2670..2744-2747..3701	95
ND1	2747..3701	33
ND1	2747..3701	40
ND1	2747..3701	46
ND1	2747..3701	102
tRNA_Gln-tRNA_Met-ND2	Complement (3768..3839)-3840..3908-3909..4950	90
ND2	3909..4950	19
tRNA_Ala-tRNA_Asn-rep origin LS-tRNA_Cys	Complement (5021..5089)-complement(5090..5163)-5164..5198-complement (5196..5262)	152
Cox1	5337..6878	28
Cox1	5337..6878	33
Cox1	5337..6878	34
Cox1	5337..6878	22
Cox1	5337..6878	23
tRNA_Ser-tRNA_Asp	Complement (6882..6950)-6954..7022	58
Cox2	7023..7706	58
Cox2	7023..7706	37
Cox2	7023..7706	18
tRNA_Lys-ATP8	7710..7775-7777..7983	44
ATP8-ATP6	7777..7983-7938..8617	37
ATP6	7938..8617	36
ATP6-Cox3	7938..8617-8618..9401	28
ND3	9471..9817	27
ND3-tRNA_Arg-ND4L	9471..9817-9818..9885-9887..10183	104
ND4	10177..11554	47
ND4	10177..11554	44
ND4	10177..11554	23

tRNA_Leu (anticodon CUN)	11683..11751	52
ND5	11752..13559	20
ND5	11752..13559	39
tRNA_Glu-Cytb	14084..14152-14156..15294	95
Cytb	14156..15294	101
Cytb	14156..15294	35
Cytb	14156..15294	41
Cytb	14156..15294	36
D-loop	15465..17131	78
**D-loop**	**15465..17131**	**200**
Total		3447

[1] Contig sequences used for phylogenetic analyses are reported in bold.

Table S2. *Prolagus sardus* mitochondrial gene and D-loop regions longer than 100 bp, obtained combing sequence information reported in Table S1.

**mtDNA region (total length)**[1]	***P. sardus* obtained sequences (bp)**[2]	**% coverage**[3]
12S (964 bp)	639	66.29
16S (1567 bp)	931	59.41
ND1 (955 bp)	237	24.82
ND4 (1377)	114	8.28
Cox2 (684 bp)	113	16.52
Cytb (1139 bp)	233	20.46
D-loop (1667 bp)	278	16.68

[1] Length of the corresponding mtDNA regions in the *Ochotona curzoniae* mtDNA is reported in brackets.
[2] Total length of the *P. sardus* combined region.
[3] Coverage of the *P. sardus* sequence on the corresponding *O. curzoniae* mtDNA region.

Table S3. Mitochondrial genome sequences of modern lagomorphs and rodent species used for the phylogenetic analyses.

**Order**	**Family**	**Species**	**Accession number**
Rodentia	Cricetidae	*Cricetulus griseus*	NC_007936.1
Lagomorpha	Ochotonidae	*Ochotona princeps*	NC_005358.1
		O. koslowi	NC_039987.1
		O. hyperborea	NC_057103.1
		O. erythrotis	MG051346.1
		O. dauurica	NC_044120.1
		O. curzoniae	NC_011029.1
		O. coreana	MT017929.1
		O. collaris	AF348080.1
	Leporidae	*Lepus yarkandensis*	MN539747.1
		L. townsendii	NC_024041.1
		L. timidus	NC_024040.1
		L. sinensis	NC_025316.1
		L. hainanus	JQ219662.1
		L. granatensis	NC_024042.1
		L. europaeus	NC_004028.1
		L. capensis	NC_015841.1
		Romerolagus diazi	MW927505.1
		Oryctolagus cuniculus	NC_001913.1

Figure S1. Calibration of the conventional radiocarbon date of the sample PR6.

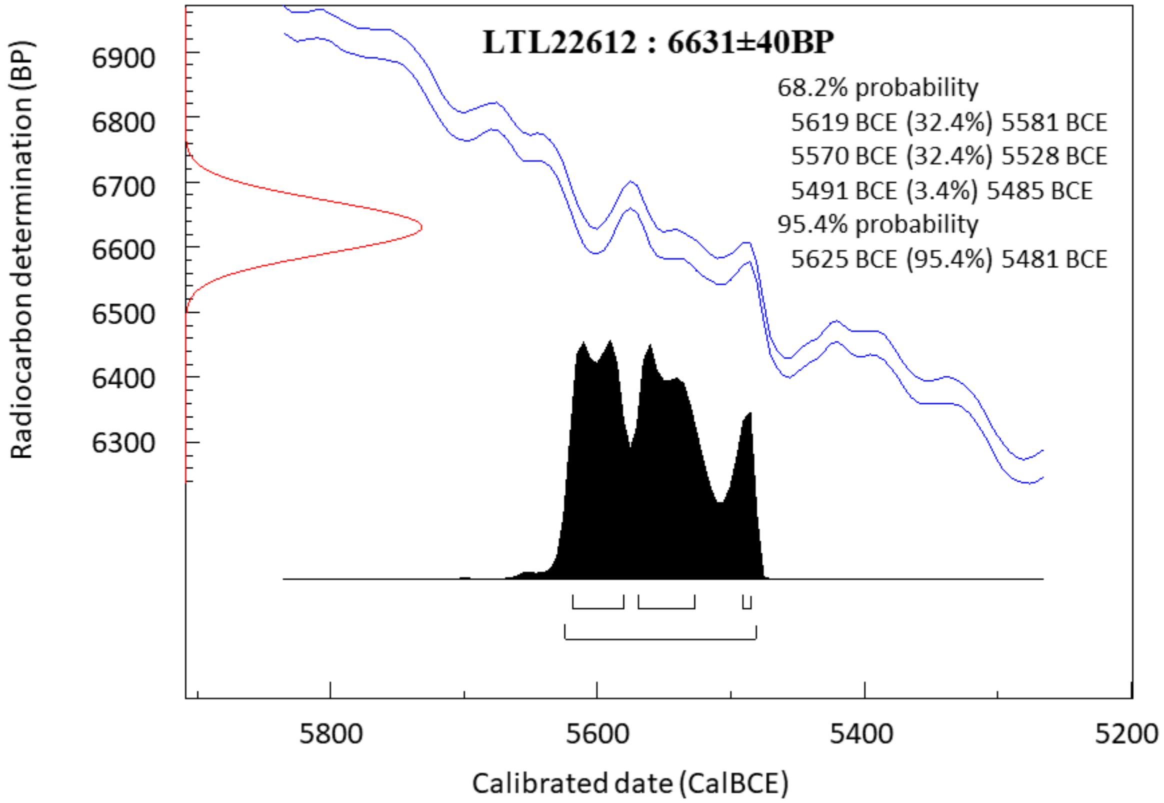

Figure S2. Nucleotide misincorporation patterns observed through mapDamage plots.

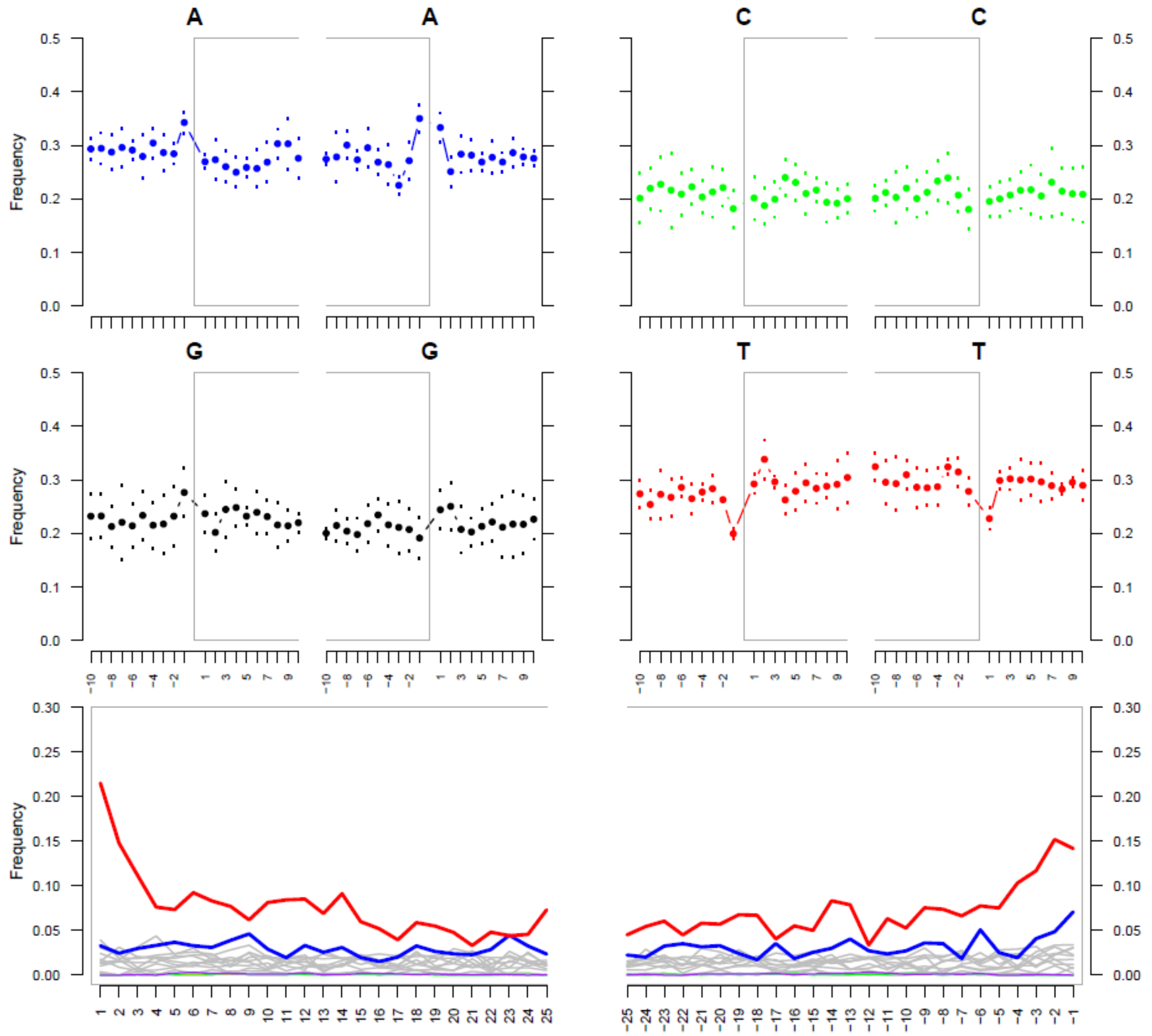

Figure S3. Mapping of the ancient mtDNA reads to the *Ochotona curzoniae* reference mtDNA sequence (NC_011029.1; 17131 bp). The vertical coverage ranged from 3X to 147X. Grey scale indicates mapping quality.

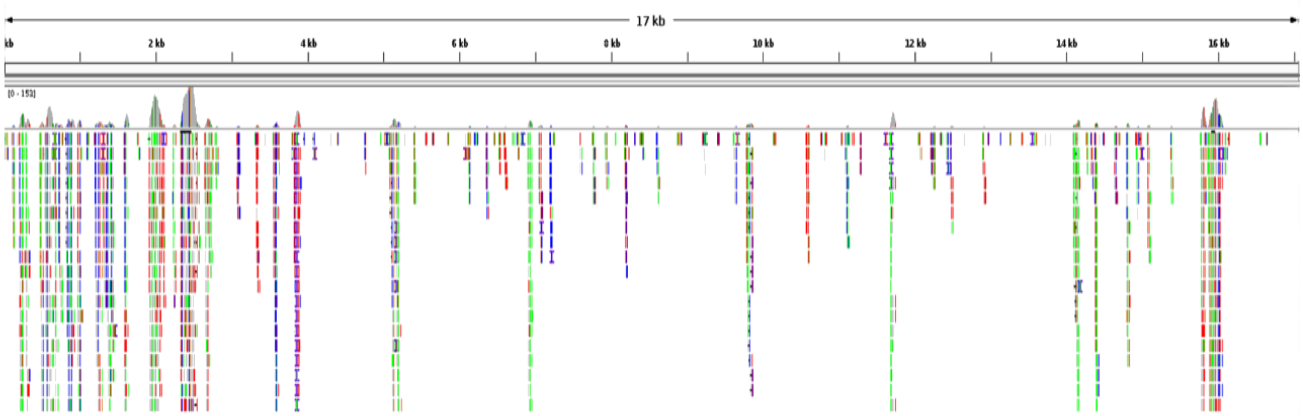

Figure S4. Timetree obtained using RelTime method implemented in Mega X software. Divergence time for the different nodes is placed close to the nodes. Two nodes are dated to set the molecular clock: the divergence node of the two Lagomorpha families (~51 Ma) and the node of the genus Ochotona (~12 Ma). Geological time is indicated by dashed lines. Pli and Quat indicate Pliocene and Quaternary eras, respectively. The geological time scale followed Gradstein *et al.* [101].

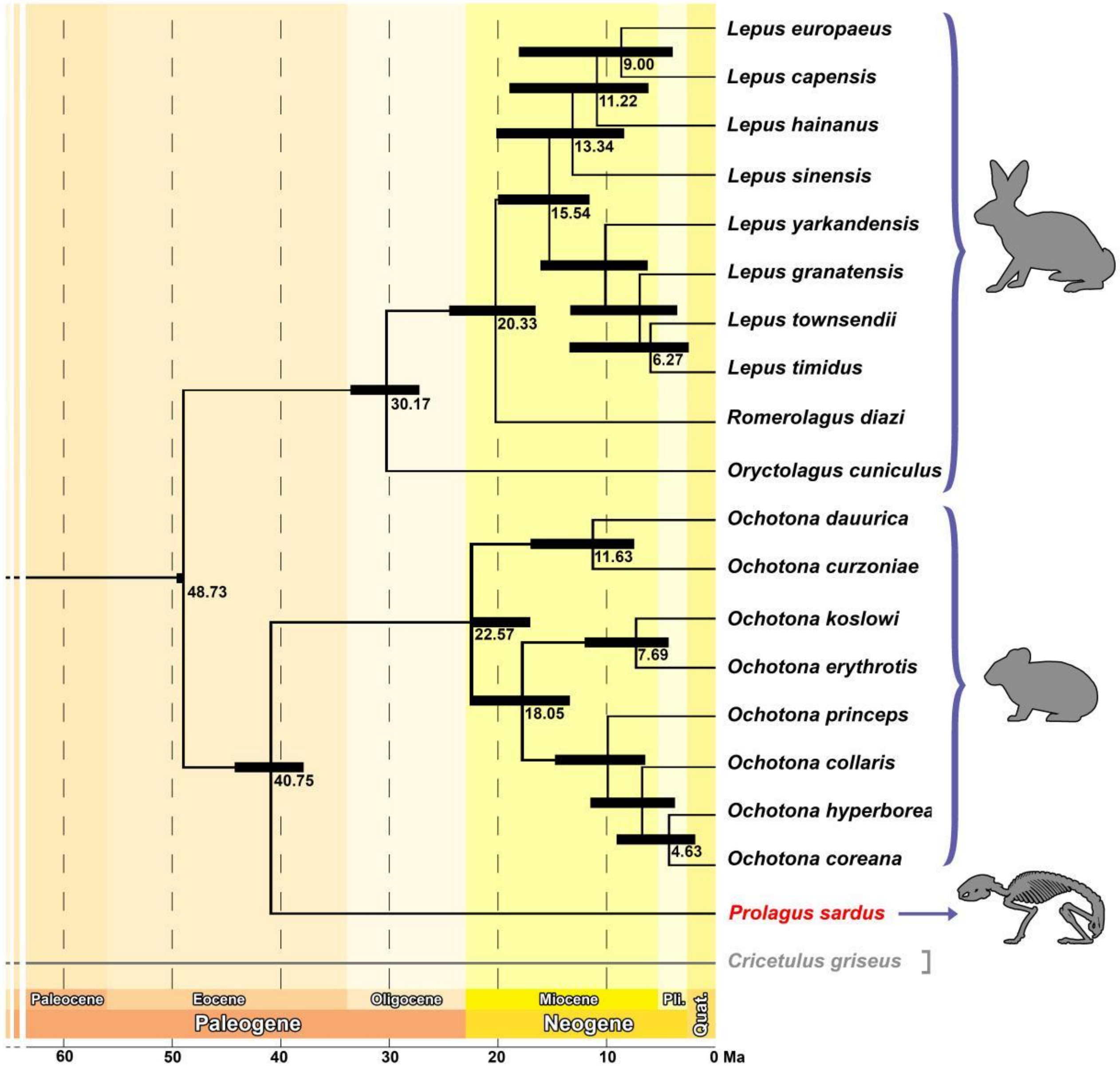

Supplement: Supplementary file 1 — Supplementary Information. [file 41598_2023_40746_MOESM1_ESM.pdf]
